# Intervention of *Uncaria* and Its Components on Liver Lipid Metabolism in Spontaneously Hypertensive Rats

**DOI:** 10.3389/fphar.2020.00910

**Published:** 2020-07-16

**Authors:** Zhenhua Tian, Shiming Zhang, Huanjuan Wang, Zhenshan Chen, Mengjia Sun, Linlin Sun, Lili Gong, Yunlun Li, Haiqiang Jiang

**Affiliations:** ^1^ Experimental Center, Shandong University of Traditional Chinese Medicine, Jinan, China; ^2^ Pharmacy School, Shandong University of Traditional Chinese Medicine, Jinan, China; ^3^ Traditional Chinese Medicine Clinical Research Base for Hypertension of Affiliated Hospital, Shandong University of Traditional Chinese Medicine, Jinan, China

**Keywords:** *Uncaria*, hypertension, lipidomics, rhynchophylline, isorhynchophylline, biomarker

## Abstract

*Uncaria rhynchophylla* (Miq.) Miq. ex Havil is widely used in the treatment of hypertension. The *Uncaria* extract and its bioactives, rhynchophylline and isorhynchophylline, reduced the blood pressure and fatty content in liver cells. In the present study, the antihypertensive effects of *Uncaria* ethanol extract (UET), rhynchophylline (RT) and isorhynchophylline (IT) were investigated in spontaneously hypertensive rats (SHR) using UPLC-Q-Orbitrap/MS based lipidomics approach. Histological changes in the liver were evaluated. Cytolysis and fatty degeneration in the liver tissues were observed in the SHR group. Lipid species in WKY, SHR treated with UET, RT, and IT were plotted to obtain the Orthogonal Projections to Latent Structures Discriminant Analysis (OPLS-DA) score plots. Fifty-six endogenous metabolites in the liver such as glycerides, glycerophospholipids, unsaturated fatty acids, and sphingomyelins were selected as potential hypertension associated biomarkers. In order to further explore the metabolite targets of UET for antihypertensive, student's t test and correlation analysis were performed to recognize the pattern recognition and to select the significant metabolites. Similar and prolonged reduction in blood pressure was observed in all SHR groups treated with UET, RT, and IT, while the metabolite profiles were perturbed slightly compared to that of the untreated SHR. Further analysis showed that only a few common components were observed in both RT and IT, which showed similar antihypertensive effect in spite of the distinct metabolic pathways. These results help in understanding the mechanisms of isomeric ingredients in exhibiting the antihypertensive effect but with different targets.

## Introduction

*Uncaria* is a traditional Chinese herbal drug that has been used to treat cardiovascular diseases such as hypertension (Heitzman et al., 2005; Zhou and Zhou, 2010). The active pharmacological components in *Uncaria* are alkaloids. The total alkaloid content in *Uncaria* is about 0.2%, in which rhynchophylline is 28–50%, isorhynchophylline is 15% (Shi et al., 2003). The extract of *Uncaria* displayed different hypotensive potencies based on the content of four constituents isorhynchophylline > rhynchophylline > total alkaloid > non-alkaloid fraction (Zhou and Zhou, 2012). The total alkaloid's hypotensive effect was due to decreased cardiac output, which results from bradcardia and not from the inhibition of cardiac contractility (Shi et al., 2003). In addition, rhynchophylline, and isorhynchophylline are a pair of diastereoisomers, which are liable to transform mutually due to their twisted conformation. From former studies, the antihypertensive effects of rhynchophylline and isorhynchophylline were found to be through inhibiting of the vasomotor center, sympathetic nerves, and blocking of L-type Ca^2+^ channels (Zhang et al., 2004; Zhou and Zhou, 2010; Li et al., 2013). The difference is that rhynchophylline has no significant effect on renal blood flow (Ndagijimana et al., 2013). Rhynchophylline has reported to ameliorate endothelial dysfunction in SHRs through the activation of Src-PI3K/Akt-eNOS signaling pathway (Hao et al., 2017). Isorhynchophylline can cause cell cycle arrest and is effective against Angiotensin II induced proliferation in rat vascular smooth muscle cells to treat cardiovascular diseases (Zhang et al., 2008). Although *Uncaria* and its components have an antihypertensive effect, different active components have different antihypertensive effects and mechanisms.

Essential hypertension is a common systemic metabolic disease caused by the interplay of multiple factors, including genetic predisposition, behavioral and environmental risk factors and other diseases (Bacon et al., 2004; Marteau et al., 2005; Urbina et al., 2008). In recent years, research has been focused on the role of lipids in hypertension (Hinterwirth et al., 2014). In our previous study, Tengfu Jiangya tablet (TJT), a traditional Chinese medicine formulation consisting of *Uncaria* and *Raphanus raphanistrum* subsp. sativus (L.) Domin, is found to increase the levels of lipid metabolites, and sphinganine levels were decreased in the model group (Tian et al., 2018). Liu et al. (2018) have proved that *Uncaria* extract's antihypertensive effect is mediated through regulation of lipid, vitamin, and amino acid metabolism.

The main organ of lipid metabolism is liver (Law et al., 2019). Excessive accumulation of triglycerides (TAGs), free fatty acids (FFAs), total cholesterol (TC), phosphatidylcholine (PC), phosphatidylethanolamine (PE), ceramide (Cer), and sphingomyelin is the main cause of lipid metabolic disorders (Kwong et al., 2015; Zhu et al., 2016; Chen et al., 2017; Walther et al., 2018). These components are mainly produced and stored in liver cells (Nguyen et al., 2008; Zhuang et al., 2015). Dysregulation of lipid metabolism is one of the main characteristics of hypertension (Graessler et al., 2009; Xie et al., 2019). Studies found that sphingolipids (Spijkers et al., 2011) and phospholipids (Biernacki et al., 2019) have significant effects on hypertension, among which is ceramide, the precursor of Sphingosine-1-phosphate (S1P), has growth-inhibiting and pro-apoptotic actions (Bourbon et al., 2002). In addition to these growth-regulating properties, researchers have shown that sphingolipids are involved in the regulation of vascular tone, for instance, by regulating nitric oxide and endothelium derive relaxing factor (EDHF) responses in different types of blood vessels (Bourbon et al., 2002; Mulders et al., 2006; Mulders et al., 2009). Fatty acids can also have an effect on hypertension. Omega-3 fatty acids are reported to possess antihypertensive effects by enhancing the release of nitric oxide (NO), as well as modifying the release of ADP, vasoactive asoactive prostanoids, and possibly endothelium-derived hyperpolarizing factor (Mori, 2006). Research shows that high levels of free fatty acids are an independent risk factor for the occurrence of hypertension (Guo et al., 2015).

Based on previous studies, we hypothesize that *Uncaria* and its components rhynchophylline and isorhynchophylline can regulate blood pressure by improving lipid and fatty acid metabolism. However, there were no reports on rhynchophylline and isorhynchophylline. Therefore, in this study, we tested the effect of these bioactive compounds and UET extract (standardized against these two bioactive compounds' concentration) on phosophoglyceride, sphingolipid, and glyceride metabolisms in spontaneously hypertensive rats using UPLC-Q-Orbitrap-MS based lipidomics analysis.

## Materials and Methods

### Chemicals and Reagents

Arachidonic acid, linoleic acid, linolenic acid, and palmitic acid (purity > 98%) were purchased from Beijing Solarbio Science & Technology Co., Ltd., China. Heptadecanoic acid (purity > 98%) was purchased from Shanghai Yuanye Bio-Technology Co., China. Palmitic acid-d31 (purity > 98%) was purchased from Sigma Company, USA. The LC-MS grade ammonium formate, methanol, dichloromethane, isopropanol, and acetonitrile were purchased from Thermo Fisher, USA. Rhynchophylline and isorhynchophylline (purity > 98%) were purchased from Shanghai Standard Technology Co., Ltd. China.

### Preparation of *Uncaria* Extract

The dried whole plant, *Uncaria rhynchophylla* (Miq.) Miq. ex Havil (No. 170906), was purchased from Huqiao Pharmaceutical Co., Ltd. (Bozhou, Anhui, China). The plant was authenticated by Professor Feng Li, School of Pharmacy, Shandong University of Traditional Chinese Medicine, Shandong. The leaves were removed from the whole plant, and the remaining plant (stem & root) was powdered and extracted with 95% ethanol under reflux for 1 h. The extract was concentrated using rotary evaporator to dryness. The extract was stored in a refrigerator at 4°C until further experimentation.

### Animals and Dosing

The animal experiment protocol was approved by the Animal Care and Ethics Committee of Shandong University of Traditional Chinese Medicine (SDUTCM2018120301). Based on previous reports (Tian et al., 2018), a total of 32 male spontaneously hypertensive rats (SHRs) and eight male Wistar Kyoto rats (WKY), weighing 200–220 g were purchased from Vital River Laboratory Animal Technology Co. Ltd., Beijing, China. All the animals were housed in an air-conditioned room at a temperature of 22 ± 2°C, 55 ± 5% humidity, and a 12 h dark/light cycle. They were given a certified standard diet and tap water *ad libitum*. The SHRs were randomly divided into four groups with eight rats in each group: the disease control group (SHR), *Uncaria*-extract-treatment group (UET, dose: 2.29 mg/kg body weight, *p.o.*, once daily for 4 weeks), rhynchophylline treatment group (RT, dose: 0.5 mg/kg body weight, *p.o.*, once daily for 4 weeks), and isorhynchophylline treatment group (IT, 0.3 mg/kg body weight, *p.o.*, once daily for 4 weeks). The WKY rats served as the normal control group (WKY, 2 ml normal saline, *p.o.*, once daily for 4 weeks). *Uncaria* extract, rhynchophylline, and isorhynchophylline were dissolved in normal saline to prepare the required concentrations for oral administration, and the maximum volume of the solution administered to the rats is not more than 2 ml.

### Measurement of Systolic and Diastolic Blood Pressure

Blood pressure of each rat was monitored on non-invasive blood pressure analysis system (Softron BP-98A, Japan) at 0, 1, 2, 3, and 4-week intervals. The results were represented as mean ± SD, and the statistical significance was analyzed by one-way analysis of variance (ANOVA) using SPSS 22.0 (SPSS Inc., USA).

### Isolation of Liver Tissues and Histology of Liver Tissues

At the end of the treatment period, the liver tissues were excised under diethylether anesthesia. The liver tissues were stored at −80°C until further experimentation.

Hematoxylin–eosin staining technique was used to observe the histological changes in rats' liver cells and tissues before and after intervention. Liver tissues were formalin-fixed and paraffin embedded, sectioned, and stained with hematoxylin and eosin (HE) by using the standard protocol (Cardiff et al., 2014). After routine processing, paraffin sections of each tissue were cut into 5–6 μm thickness and stained with hematoxylin and eosin. The stained slices were examined under an optical microscope (Zeiss Axioscope) at 200× magnification (Do et al., 2012).

### Preparation of Fatty Acid Standards

Palmitic acid-d_31_ [17:1/12:0] (1.65 mg/ml) was used as an internal standard for adjustment of possible inter- and intra-assay variances. Fatty acid standards; arachidonic acid (1.27 mg/ml), linoleic acid (2.16 mg/ml), linolenic acid (1.39 mg/ml), palmitic acid (1.52 mg/ml), and heptadecanoic acid (1.48 mg/ml) were used as standards to identify the fatty acids. All fatty acids were dissolved in 1 ml of HPLC grade n-hexane.

### Preparation of Liver Extracts for Fatty Acid Analysis

Liver samples (about 50 mg each) containing 100 µl of internal standard solution were homogenized in 400 µl isopropanol containing 2% phosphate buffer (2 M) for 1 min. The tissue homogenates were centrifuged at 11,000× g for 10 min at 4°C. To the supernatant solution (450 µl), a mixture of isopropanol (800 μl) and water (300 μl) was mixed and vortexed for 2 min. The solution was centrifuged for another 10 min at 11,000×g at 4°C, and the supernatant (800 μl) was dried under nitrogen stream. The dried residue was dissolved in 1,400 μl methanol (LC-MS grade) for the analysis of fatty acids.

### Preparation of Liver Extracts for Lipid Analysis

Lipids were extracted from the liver samples using a modified version of Folch method (Iverson et al., 2001). Briefly, liver tissues (about 50 mg each) were homogenized in 0.5 ml of cold double distilled water followed by the addition of 1.2 ml dichloromethane:methanol (2:1 v/v) (Ubhi, 2018). The mixture was centrifuged at 11,000 g at 4°C for 15 min. The dichloromethane fraction of the supernatant was carefully collected and evaporated to dryness. The dried residue was dissolved in a mixture of isopropanol:acetonitrile:water (2:1:1) for the analysis of lipids.

### UPLC-ESI-MS/MS Conditions

Q-Exactive Orbitrap mass spectrometer (Thermo Fisher Scientific) was connected to an UltiMate 3000 UPLC *via* an electrospray ionization ion (ESI) source for the fatty acid and lipid analysis. The chromatographic conditions were shown in **Table 1**.

**Table 1:** Chromatographic and mass spectrometric parameters for lipids and fatty acids.

	Lipid metabolites	Fatty acid metabolites
Column	Halo C_18_ (100 × 2.1 mm, 2.7 μm)	Halo C_18_ (100 × 2.1 mm, 2.7 μm)
Mobile phase	A: ACN/H_2_O (4:6) with 10 mM HCOONH_4_ B: ACN/isopropanol (1:9) with 10 mM HCOONH_4_	A: H_2_O B: ACN/isopropanol (8:2)
Gradient	15–40% B 0–5 min 40–70% B 5–5.5 min 70–75% B 5.5–7.5 min 75–78% B 7.5–15 min 78–85% B 15–24 min	70–70% B 0–2 min 70–75% B 2–5 min 75–80% B 5–10min 80–90% B 10–13 min 90–99% B 13–16 min
Flow rate	300 μl/min	400 μl/min
Injection Volume	5 μl	1 μl
ESI parameters polarity	positive and negative	negative
Column temp	45°C	40°C
auxiliary gas flow rate	10 arbitrary units)	10 arbitrary units
spray voltage	3,500 V (positive) 2,800 V (negative)	3,000 V
auxiliary gas temperature	350°C (positive) 320°C (negative)	350°C
Ion source temp	350°C (positive) 300°C (negative)	300°C
Scanning range	200–2,000 m/z	60–1050 m/z
(S)-lens RF level	55	55
resolution	70,000	70,000
Normalized Collision Energy	20, 50, 70 eV	20, 50, 70 eV

### Data Processing and Identification of Biomarkers

The data was processed as described in the literature (Feng et al., 2019). Simca-P 14.1 software (Umetrics AB, Umea, Sweden) was used for principal component analysis (PCA) and Orthogonal Projections to Latent Structures Discriminant Analysis (OPLS-DA). The fatty acid and lipid species were identified from the PCA and OPLS-DA loading plots. The differential expression of fatty acid and lipid biomarkers in SHR compared to those in WKY rats was identified from Variable Importance in Projection (VIP) values of greater than 1. The fold change was calculated using Mass Profiler Professional software, Agilent Technologies. The statistical significance was calculated using student t-test. The biomarkers with P value less than 0.05 and the fold change greater than 2 were considered differentially regulated in SHR compared to WKY rats. The differentially regulated biomarkers were identified by searching HMDB (http://www.hmdb.ca/), LIPID MAPS Lipidomics Gateway (http://www.lipidmaps.org) and METLIN (https://metlin.-scripps.edu) databases. The web-based tools, KEGG (http://www.genome.jp/kegg/) and Metaboanlayst (https://www.metaboanalyst.ca/) were used for pathway analysis and visualization of data.

## Results

### Effect of UET Standardized Extract, Rhynchophylline, and Isorhynchophylline on Blood Pressure

**Figure 1:**
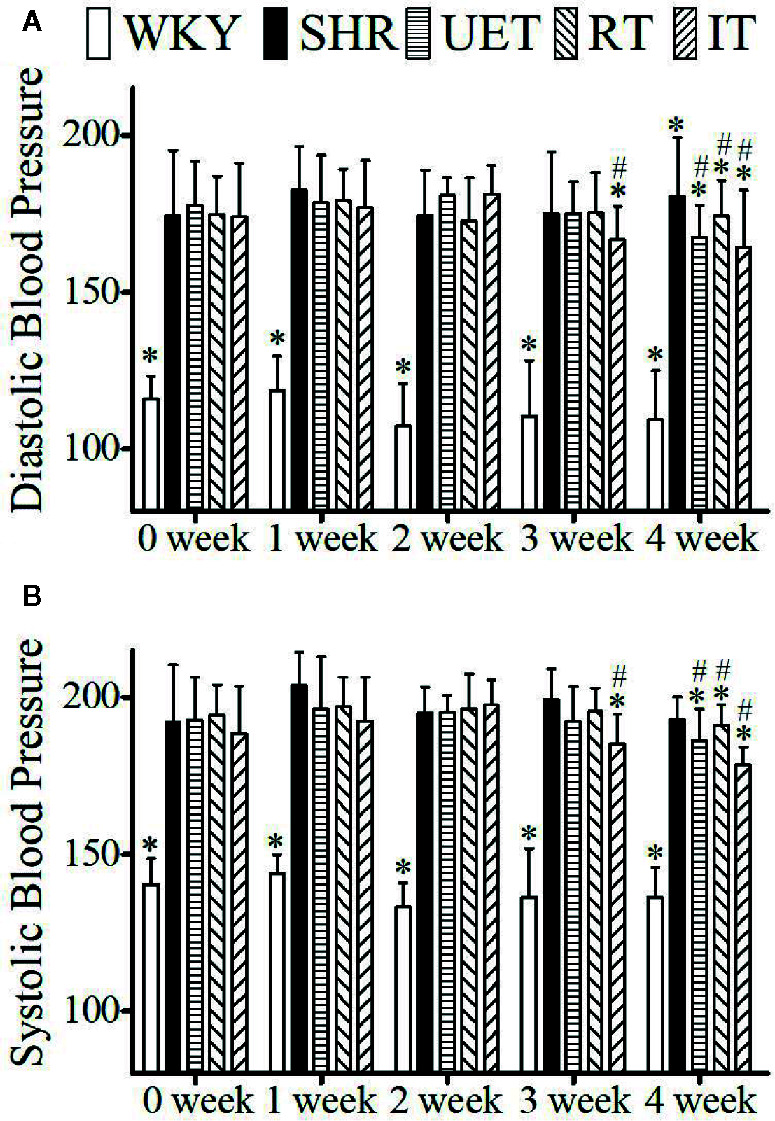
The effect of WKY, SHR, UET, RT, and IT on systolic and diastolic blood pressure in spontaneously hypertensive rats. **(A)** Time-dependent changes in systolic blood pressure in WKY, SHR, UET, RT, and IT. **(B)** Time-dependent changes in diastolic blood pressure in WKY, SHR, UET, RT and IT (*P < 0.05 compared with SHR, ^#^P < 0.05 compared with 0 week).

The effect of *Uncaria*-extract, rhynchophylline, and isorhynchophylline on systolic and diastolic blood pressure is shown in **Figure 1**. Compared to WKY, the systolic and diastolic blood pressure in SHR was significantly high. The mean systolic and diastolic blood pressure in WKY were 137.83 ± 10.5 and 111.50 ± 14.18 mm of Hg respectively while they were 196.10 ± 11.06 and 178.54± 15.21 mm of Hg respectively in the SHR group. Throughout the experiment, there was no significant change in either WKY or SHR. Treatment with *Uncaria*-extract, rhynchophylline, and isorhynchophylline significantly reversed the elevated levels of blood pressure in SHR, and the effect was found to be time-dependent.

### Effect of UET Standardized Extract, Rhynchophylline, and Isorhynchophylline on Liver Histology

**Figure 2:**
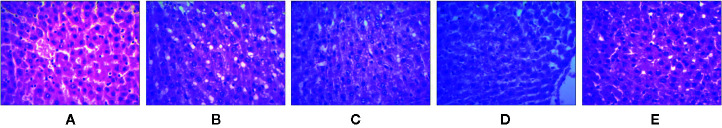
The liver tissue sections of spontaneously hypertensive rats were stained with H&E. **(A)** WKY group, **(B)** SHR group, **(C)** UET group, **(D)** RT group, **(E)** IT group.

The effect of test compounds on liver histology is illustrated in **Figure 2**. In the WKY group, the hepatocytes were found to be normal and nuclear structure was clear. In the SHR group, the hepatocytes were found to be enlarged with cytolysis and fatty degenerations. In SHR treated with test compounds, the hepatocyte was found to be normal and either cytolysis or fatty degeneration was not observed indicating that the test compounds reversed the pathological changes in SHR.

### UPLC-Q/Orbitrap-MS Method Validation

The possible mechanism of action of test compounds was determined using lipidomics analysis of liver homogenates using UPLC-Q-Orbitrap/MS. Total ion chromatograms (TICs) of lipid extracts from liver homogenates showed good separation (**Figure S1**).

To confirm the significant difference in liver metabolites observed in LC-MS originated from inherent difference between groups rather than from the instrumental drift, the instrument stability and reproducibility were analyzed using quality control (QC) samples as described elsewhere in the literature (Qin et al., 2018). The quality control parameters were shown in the supplementary information (**Table S1** and **Figures S2** and **S3**).

### Identification of Potential Biomarkers for Hypertension

Raw data were converted into mzXML format by ProteoWizard and processed with XCMS (http://metlin.scripps.edu/download/) for peak recognition, alignment, and correction. The parameters used were default settings except for the following: ppm = 10, bw = 10, and snthresh = 20. A visual data matrix containing retention time, m/z pairs, sample names, and normalized ion intensities was generated and exported to Simca-P 14.1 software for multivariate data analysis (Tian et al., 2018).

**Figure 3:**
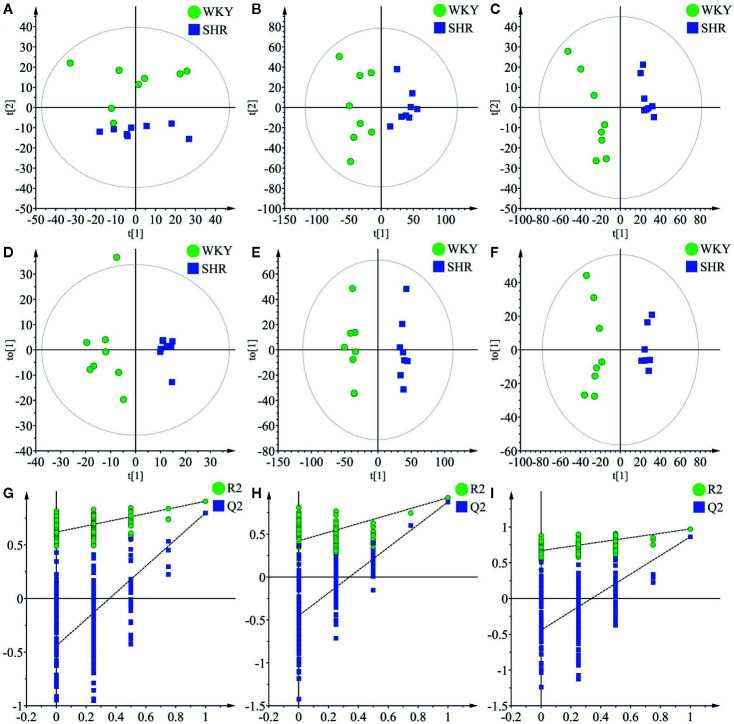
Multivariate data analysis of liver lipidomics. **(A)** The PCA score plots of fatty acid in negative mode (R^2^X = 0.801, Q^2^^=^ 0.588). **(B)** The PCA score plots of lipid species in positive mode (R^2^ = 0.579, Q^2^ = 0.239). **(C)** The PCA score plots of lipid species in negative mode (R^2^ = 0.709, Q^2^ = 0.375). **(D**) OPLS-DA score plots of fatty acid in negative mode (R^2^X = 0.409, R^2^Y = 0.906, Q2 = 0.798). **(E)** OPLS-DA score plots of lipid species in positive mode (R^2^X = 0.327, R^2^Y = 0.923, Q^2^ = 0.875). **(F)** OPLS-DA score plots of lipid species in negative mode (R^2^X = 0.598, R^2^Y = 0.989, Q^2^ = 0.863). **(G)** Permutation test of fatty acid OPLS-DA model in negative mode (R^2^ = 0.619 and Q^2^ = −0.442). **(H)** Permutation test of lipid species OPLS-DA model in positive mode (R^2^ = 0.422 and Q2 = −0.451). **(I)** Permutation test of lipid species OPLS-DA model in negative mode (R^2^ = 0.670 and Q^2^ = −0.443).

Principal component analysis (PCA) was performed to determine the differential regulation of fatty acid and lipid species in SHR compared to WKY, and the results were shown as score plots. The score plot of fatty acids in negative ionization mode was shown in **Figure 3A**. The score plots of lipid species in positive and negative ionization modes were shown in **Figure 3B** and **Figure 3C**, respectively. The perturbations in fatty acids and lipid species in SHR compared to WKY were clearly revealed in the score plots. The model parameters (R^2^X) representing the explanative ability of the model shown in **Figures 3A–C** were found to be 0.801, 0.579, and 0.709, respectively.

OPLS-DA was carried out to determine the changes in potential biomarkers in SHR compared to WKY. The score plot of fatty acids in negative ionization mode was shown in **Figure 3D** (R^2^X = 0.409, R^2^Y = 0.906, Q^2^ = 0.798). The score plots of lipid species in negative and positive ionization modes were shown in **Figure 3E** (R^2^X = 0.327, R^2^Y = 0.923, Q^2^ = 0.875) and **Figure 3F** (R^2^X = 0.598, R^2^Y = 0.989, Q^2^ = 0.863) respectively.

Permutation testing and cross validation, two established methods of internal validation, were used to confirm model validity (Rubingh et al., 2006). Permutation tests involve the random assignment of class labels to cases and controls. Permutation testing using 100 random permutations demonstrates that the goodness of fit and predictive ability (R^2^/Q^2^) of the SHR group discriminating WKY group (**Figure 3**). The score plots were found to be reliable and robust without overfitting (Davis et al., 2012; Ma et al., 2017).

Variable importance for projection (VIP) values produced in OPLSDA were applied to find potential biomarkers and variables with VIP > 1 were further processed by Mass Profiler Professional (Agilent Technologies, USA) for Student's t-test and fold change (FC) (Davis et al., 2012; Kwan et al., 2013). The biomarkers with VIP values >1, P value less than 0.05 and FC value greater than 2 were considered significant in SHR compared to those in WKY.

The metabolites were identified based on molecular ion peaks ([M+H]^+^, [M−H]^−^ *etc.*) and mass fragment (MS^2^) ions in addition to comparing the retention times with the metabolites in the human metabolome database (HMDB, http://www.hmdb.ca), Lipidomics Gateway (http://www.lipidmaps.org), METLIN (https://metlin.-scripps.edu) and KEGG (http://www.genome.jp/kegg/) database. The pathway analysis was performed using MetaboAnalyst 4.0 software (http://www.metaboanalyst.ca). Library search was performed using a maximum mass deviation of 5 ppm. In addition to comparing the mass fragment patterns with those reported in these databases (Milne et al., 2006), the identity of the lipid metabolites was confirmed by comparing with commercially available authentic standards (Zhang et al., 2019). The mass fragment patterns of fatty acid metabolites were shown in the supplementary material (**Figure S4**), and the m/z values of the metabolites were shown in **Table 2**.

**Table 2:** 56 identified potential biomarkers among the WKY, SHR and test compounds.

No	Fragment	tR(min)	m/z	Change				Biomarkers
				^a^SHR	^b^UET	^b^RT	^b^IT	
1	[M−H]^−^	9.46	315.25430	↓	↑		↑	MG(15:0)
2	[M+H]^+^	12.57	595.52951	↑				DG(34:1)
3	[M+H]^+^	11.23	593.51672	↑	↓			DG(34:2)
4	[M+H]^+^	11.23	611.54452	↑	↓		↓	DG(35:0)
5	[M+H]^+^	9.22	607.51278	↑	↓			DG(35:2)
6	[M+H]^+^	11.30	621.53383	↑				DG(36:2)
7	[M+H]^+^	11.31	619.53112	↑				DG(36:3)
8	[M+H]^+^	10.20	617.51453	↑			↓	DG(36:4)
9	[M+H]^+^	10.20	635.54441	↑	↓		↓	DG(37:2)
10	[M+H]^+^	21.09	819.72638	↑				TG(49:1)
11	[M+H]^+^	21.14	845.74194	↑				TG(51:2)
12	[M+H]^+^	21.28	859.75805	↑			↓	TG (52:2)
13	[M+H]^+^	21.42	855.74175	↑				TG (52:4)
14	[M+H]^+^	20.94	873.69390	↑				TG (54:9)
15	[M+H]^+^	20.83	893.74210	↑	↓		↓	TG (55:6)
16	[M+H]+	21.65	919.85124	↑				TG (56:0)
17	[M+H]^+^	20.51	897.69435	↑	↓		↓	TG (56:11)
18	[M+H]^+^	21.41	911.78964	↑	↓		↓	TG (56:4)
19	[M+H]^+^	21.25	941.83068	↑	↓		↓	TG (58:0)
20	[M+H]^+^	20.07	921.69465	↑	↓		↓	TG (58:13)
21	[M−H]^−^	3.41	423.27580	↓		↑		LysoPA (18:0)
22	[M−H]^−^	6.16	465.30469	↓	↑	↑	↑	LysoPA (20:0)
23	[M+H]^+^	5.89	522.35524	↓				LysoPC (18:1)
24	[M+H]^+^	5.37	520.33999	↓				LysoPC (18:2)
25	[M+H]^+^	6.65	552.40265	↓				LysoPC (20:0)
26	[M+H]^+^	5.33	546.34363	↓				LysoPC (20:3)
27	[M+H]^+^	5.32	544.33956	↓				LysoPC (20:4)
28	[M+H]^+^	6.65	578.41803	↓				LysoPC (22:1)
29	[M+H]^+^	7.16	606.45261	↓	↑			LysoPC (24:1)
30	[M-H]^-^	5.01	480.30993	↓				LysoPE (18:0)
31	[M+H]^+^	4.81	476.27854	↓			↑	LysoPE (18:3)
32	[M+H]^+^	6.05	510.35628	↓				LysoPE (20:0)
33	[M+H]^+^	4.59	498.26282	↓			↑	LysoPE (20:5)
34	[M-H]^-^	16.07	661.50530	↑	↓	↓	↓	PA (34:0)
35	[M+H]^+^	12.95	832.66404	↓	↑			PE (32:0)
36	[M+H]^+^	8.59	702.50617	↓				PE (33:2)
37	[M+H]^+^	10.24	802.62359	↓				PE (40:1)
38	[M+H]^+^	10.24	800.61666	↓				PE (40:2)
39	[M+H]^+^	15.32	856.68942	↓	↑		↑	PE (44:1)
40	[M+H]^+^	6.61	815.48703	↑				PG (40:9)
41	[M+H]^+^	7.22	799.56799	↑				PG (38:4)
42	[M+H]^+^	6.65	821.52288	↑				PG (40:7)
43	[M+H]^+^	6.60	863.55928	↑				PI (36:2)
44	[M+H]^+^	6.59	840.57451	↑			↓	PS (40:4)
45	[M+H]^+^	10.27	677.56590	↑	↓		↓	SM (d18:0/14:0)
46	[M−H]^−^	12.68	298.28348	↓				Sphingosine
47	[M−H]^−^	4.34	277.21738	↓	↑	↑	↑	*α*-linolenic acid
48	[M−H]^−^	12.21	335.29599	↓				Docosadienoate (22:2)
49	[M−H]^−^	2.12	299.25947	↑				Hydroxystearic acid
50	[M−H]^−^	16.10	279.23315	↑	↓	↓		Linoleic acid
51	[M−H]^−^	0.51	181.03348	↑				Methyluric acid
52	[M−H]^−^	12.67	297.28013	↓				Pristanic acid
53	[M−H]^−^	6.67	355.26473	↓				Tetracosahexaenoic acid
54	[M−H]^−^	9.87	296.26777	↓				Palmitoleoyl Ethanolamide
55	[M−H]^−^	11.7	593.54197	↓	↑			CE(14:1)
56	[M−H]^−^	0.52	259.02717	↑		↓		Glucose 1-phosphate

(↑): upregulated (p < 0.05, n = 8), (↑): downregulated (p < 0.05, n = 8).

^a^Trends of the SHR group compared with the WKY group of metabolites. ^b^Trends of the vtest compounds compared with the SHR group of metabolites.

A total of 56 lipid species were identified and shown in **Table 2**. In the SHR group, compared with WKY, the concentrations of DG (34:1, 34:2, 35:0, 35:2, 36:2, 36:3, 36:4, 37:2), TG (49:1, 51:2, 52:2, 52:4, 54:9, 55:6, 56:0, 56:11, 56:4, 58:0, 58:13), PA (34:0), PG (38:4, 40:7, 40:9), PI (36:2), PS (40:4), SM (d18:0/14:0), Hydroxystearic acid, Linoleic acid, Methyluric acid, and Glucose 1-phosphate were significantly increased (P<0.05), while LysoPA (18:0, 20:0), LysoPC (18:1, 18:2, 20:0, 20:3, 20:4, 22:1, 24:1), LysoPE (18:0, 18:3, 20:0, 20:5), MG (15:0), PE (32:0, 33:2, 40:1, 40:2, 44:1), Sphingosine, *α*-linolenic acid, Docosadienoate (22:2), Pristanic acid, Tetracosahexaenoic acid, Palmitoleoyl Ethanolamide, CE(14:1) were decreased.

**Figure 4:**
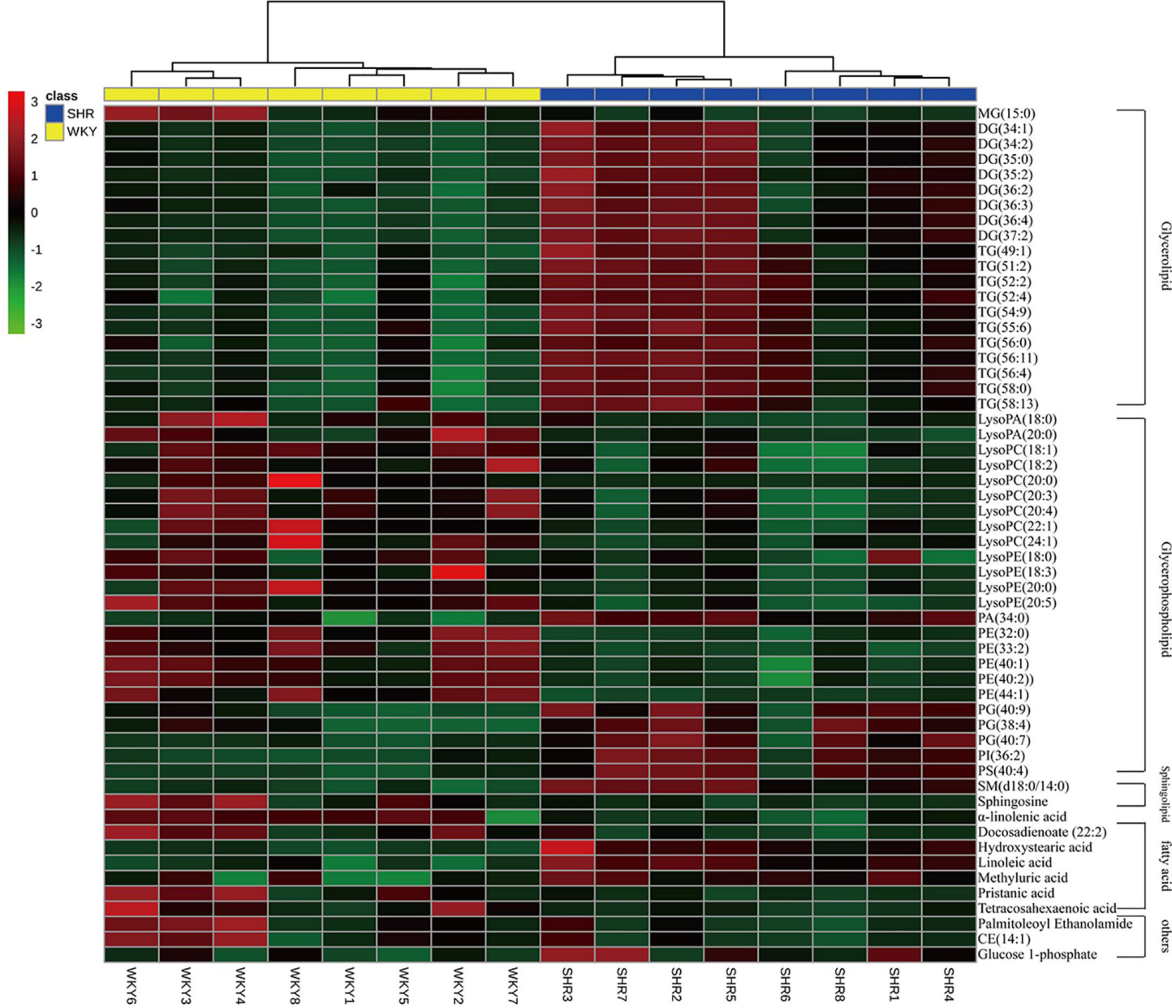
Heatmap of 56 lipid species among WKY and SHR group. Each line of this graph represents an accurate mass ordered by retention time, colored by its abundance intensity. The scale from −3 green (low abundance) to +3 red (high abundance) represents the abundance, respectively.

To further understand the metabolic differences between WKY and SHR, the identified lipid data were analyzed using clustering heatmap (Miao et al., 2015). The identified lipids clearly distinguish the metabolic profile of the SHR and as such can be regarded as potential biomarkers (**Figure 4**). Heatmap directly showed the variation of each fatty acid and lipid species and the identified compounds were visualized in a clustering heat map which illustrates the relative increase (red) or decrease (green) of values in the hyperlipidemic compared with WKY and shown in **Figure 4**.

Student t test was used to determine the significance of the hypertension-related biomarkers regulated by the test compounds in SHR. According to the resultsshown in Table 2, UET significantly reversed the following biomarkers in SHR: DG (34:2, 35:0, 35:2, 37:2), TG (55:6, 56:11, 56:4, 58:0, 58:13), LysoPA (20:0), LysoPC (24:1), MG (15:0), PA (34:0), PE (32:0, 44:1), SM (d18:0/14:0), a-linolenic acid and linoleic acid, CE (14:1); RT significantly reversed the following biomarkers in SHR: LysoPA (18:0, 20:0), PA (34:0), a-linolenic acid, linoleic acid, glucose 1-phosphate; IT significantly reversed the following biomarkers in SHR: MG (15:0), DG (35:0, 36:4, 37:2), TG (52:2, 56:6, 56:11, 56:4, 58:0, 58:13), LysoPA (20:0), LysoPE (18:3, 20:5), PA (34:0), PE (44:1), PS (40:4), SM (d18:0/14:0), a--linolenic acid.

### Pathway Impact Analysis of Hypertension-Related Metabolites

**Figure 5:**
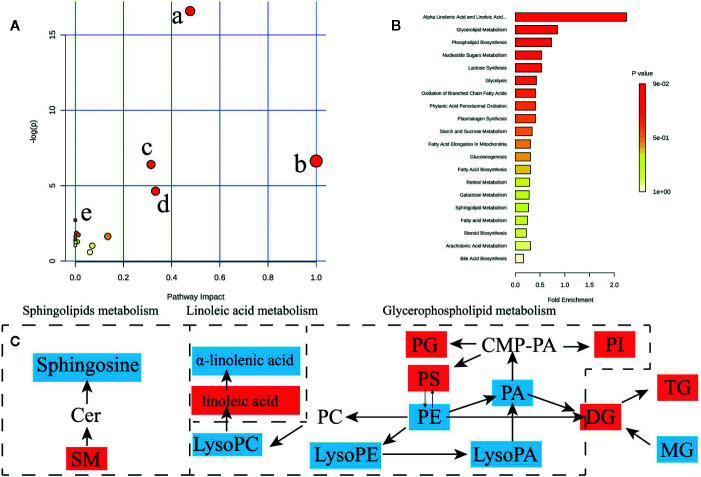
Lipid and fatty acid metabolic pathway analysis of identified differential lipid species. **(A)** Summary of IPA with MetPA including, (a) glycerophospholipid metabolism, (b) Linoleic acid metabolism, (c) sphingolipid metabolism, (d) alpha-Linolenic acid metabolism, (e) biosynthesis of unsaturated fatty acids from significantly differential fatty acid species and lipid species. The size and color of each circle are based on pathway impact value and p value, respectively. **(B)** QEA performed using MSEA. **(C)** The network of the potential biomarkers variation for SHRs compared with WKY. Red, upregulated biomarkers; blue, down-regulated biomarkers.

To determine possible metabolic pathways and networks influenced by hypertension, IPA was performed with Metabolomics Pathway Analysis (MetPA), a web-based tool for pathway analysis and visualization of metabolomics (Miao et al., 2016). A total of 56 identified metabolites were mapped to KEGG metabolic pathways for over-representation and pathway topology analyses. The differential lipid species were analyzed by MetPA and the results were shown in **Table 3**. The impact-value threshold was set to 0.01, and the pathway with impact-value above this threshold was filtered out. Ultimately, the IPA revealed dysregulation of 18 in spontaneously hypertensive rats (**Figure 5A** and **Table S2**). The pathway impact factor was evaluated by the relative importance of the compounds. **Figure 5** showed that Alpha Linolenic Acid and Linoleic Acid Metabolism, Glycerolipid Metabolism, and Phospholipid Biosynthesis had the highest impact factors. In addition, 18 metabolic pathways were found to be dysregulated in spontaneously hypertensive rats on the basis of the analysis of the quantitative enrichment analysis (QEA) algorithm of the (metabolomics pathway analysis) MSEA method (**Figure 5B**) and overview of the integrated metabolic pathway.

**Table 3:** Ingenuity pathway analysis with MetPA from differential fatty acid and lipid species.

Pathway name	Total metabolites	Hits	p	-log(p)	Holm p	FDR	Impact	KEGG
Glycerophospholipid metabolism	36	7	0.000	16.578	0.000	0.000	0.477	00564
Linoleic acid metabolism	5	2	0.001	6.633	0.109	0.046	1.000	00591
Sphingolipid metabolism	21	3	0.002	6.402	0.136	0.227	0.314	00600
alpha-Linolenic acid metabolism	13	2	0.010	4.635	0.786	0.38305	0.333	00592
Biosynthesis of unsaturated fatty acids	36	2	0.067	2.708	1.000	1.000	0.000	01040
Glycosylphosphatidylinositol (GPI)-anchor biosynthesis	14	1	0.155	1.863	1.000	1.000	0.004	00563
Glycerolipid metabolism	16	1	0.175	1.740	1.000	1.000	0.012	00561
Pentose and glucuronate interconversions	18	1	0.195	1.633	1.000	1.000	0.000	00040
Starch and sucrose metabolism	18	1	0.195	1.633	1.000	1.000	0.135	00500
Propanoate metabolism	23	1	0.243	1.416	1.000	1.000	0.000	00640
Glycolysis Gluconeogenesis	26	1	0.270	1.310	1.000	1.000	0.000	00010
Galactose metabolism	27	1	0.279	1.277	1.000	1.000	0.010	00052
Phosphatidylinositol signaling system	28	1	0.288	1.246	1.000	1.000	0.002	04070
Arachidonic acid metabolism	36	1	0.354	1.038	1.000	1.000	0.000	00590
Amino sugar and nucleotide sugar metabolism	37	1	0.362	1.016	1.000	1.000	0.071	00520
Purine metabolism	66	1	0.555	0.589	1.000	1.000	0.061	00230

(“Total” is the total number of differential lipid species in the pathway; “hits” is the actually matched number from the user differential fatty acid and lipid species; the raw p is the original p calculated from the enrichment analysis; the “Holm p” is the p value adjusted by Holm−Bonferroni method; “impact” is the pathway impact value calculated from pathway topology analysis).

## Discussion

The effect of UET standardized extract, rhynchophylline, and isorhynchophylline on hypertension was evaluated in spontaneously hypertensive rats (SHR) model. The results showed that there is upregulation of 30 and down regulation of 26 liver lipid species and fatty acids in SHR compared to WKY. As reported in various metabolome databases (Lam et al., 2017; Ma et al., 2017) these dysregulated lipid species and fatty acids are associated with hypertension and mediated through glycerophospholipid metabolism and fatty acid biosynthesis. The UET reversed the levels 19 metabolites while RT and IT reversed 6 and 18 respectively in SHR. In addition, lipid pathway analysis revealed that UET showed antihypertensive effect through alteration of glycerophopholipid, linoleic acid, and sphingolipid metabolic pathways. RT's antihypertensive effect was found to be mediated through regulation of the fatty acid pathway. IT's antihypertensive effect was found to be mediated through glyceride and phosopholipid pathway.

### Effects of UET and IT on Hepatic Sphingomyelin

In this study, it is found that sphingomyelin SM (d18:0/14:0) level was increased in SHR compared to that in WKY. The increased sphingomyelin content is reported to reduce endothelial membrane fluidity. In our study, the treatment with UET and IT significantly decreased the elevated SM (d18:0/14:0) levels in SHR (Dorrance et al., 2001).

### Effects of UET, RT, IT on Hepatic Fatty Acid

The levels of linoleic acid, hydroxystearic acid, and pristanic acid were increased while *α*-linolenic acid levels were decreased in SHR compared to WKY. Treatment with UET and RT reversed both *α*-linolenic acid and linoleic acid levels, while IT reversed only *α*-linolenic acid. All the test compounds do not have any effect on saturated fatty acids, hydroxysteraic acid, and pristanic acid.

### Effects of UET on Hepatic LysoPCs

LysoPC belongs to the group of glycerophospholipids. LysoPC is relatively abundant in blood of humans and animals (150–350 μM) and is known as the major component of oxidized low-density lipoproteins (Aoki et al., 2002). Currently, some reports demonstrated the relationship between LysoPC and blood pressure (Matsutomo et al., 2017). Hirayama et al. showed dose-dependent vasorelaxant effect of LysoPC in the rat mesenteric artery (Hirayama et al., 1998). This vasorelaxation involves increased NO production since the effect was inhibited by NO synthase inhibitor and platelet activating factor (PAF) receptor antagonist. An *in vivo* study also showed that LysoPC increased coronary blood flow, decreased coronary and renal vascular resistance, and reduced mean arterial pressure, demonstrating the vasorelaxant effect of LysoPC (Wolf et al., 1991). In this study, UET treatment reversed LysoPCs (18:2, 20:3, 20:4, 24:1). Our findings suggest that LysoPCs which have fatty acids with specific number of carbon atoms and double bonds may have a critical involvement in the regulation of blood pressure and UET and IT treatment lowered blood pressure in SHR by altering their liver lysoPCs levels.

### Effects of UET and IT on Hepatic Triglycerides, Diglycerides Metabolism

According to a study conducted by Hu et al. (2010), triglycerides demonstrate lipotoxic effects. Subsequently, it was found that patients suffering from hypertension are associated with the build-up of triglycerides in the blood plasma. As found in this study, it was also shown that antihypertensive drugs prescribed now-a-days only showed moderate effects in hypertensive patient's lipid level modification (Hu et al., 2011). Kulkarni et al. (2013) pointed out that DG (16:0/22:5), DG (16:0/22:6), and PE (40:6) are closely related to hereditary hypertension. Hu et al. (2011) found that plasma TG (C48, C50, C52, C54, C56) showed an upward trend in patients with hypertension. It was found that the levels of TG (C53, 54, 56, 58, 60, 66) in the liver of hypertensive patients were higher than those of normal subjects. In our study, it was found that the levels of DG (34:1, 34:2, 35:0, 35:2, 36:2, 36:3, 36:4, 37:2), TG (49:1, 51:2, 52:2, 52:4, 54:9, 55:6, 56:0, 56:11, 56:4, 58:0, 58:13) in the SHR group were significantly higher as compared to those in WKY group, suggesting that hypertension is related to DG and TG accumulation in the liver. UET regulated the metabolism of DG (34:2, 35:0, 35:2, 36:4, 37:2) and TG (54:9, 55:6) in spontaneously hypertensive rats. IT regulated the metabolism of MG (15:0), DG (35:0, 36:4, 37:2) and TG (52:2, 56:6, 56:11, 56:4, 58:0, 58:13) in spontaneously hypertensive rats.

## Conclusions

*Uncaria* ethanolic extract and its bioactive constituents rhynchophylline and isorhynchophylline have shown similar antihypertensive effects *in vivo*. UPLC-Q-Orbitrap-MS based lipidomics analysis on combined with pathway analysis strategy was successfully established to investigate the metabolic phenotypes of *Uncaria* extract, rhynchophylline, and isorhynchophylline-treated SHR. The study revealed the lipid-regulation characteristics of SHRs with UET, RT, and IT, which indicated a weak regulatory effect of UET, RT, and IT on the lipid disorder. In addition, the regulatory effects of the test compounds on the lipid profiles and fatty acid were different. These results may provide useful information in understanding the possible antihypertensive mechanisms of the different compounds in *Uncaria*. A similar antihypertensive effect could be produced by the RT and IT, whereas this similarity might be circumstantial and caused by different metabolic pathways due to RT and IT. The results showed RT can regulate six hypertension related metabolites, which are mainly glycerophospholipids and unsaturated fatty acids. IT can regulate 18 kinds of hypertension-related metabolites, mainly glycerides. It was also found that *Uncaria* ethanol extract demonstrated antihypertensive activity highly similar to both isomeric ingredients rhynchophylline and isorhynchophylline. Hence, this proves that the isomeric ingredients exhibit the same antihypertensive activity but with different targets, and our study provides a promising strategy to elucidate the underlying mechanisms of this phenomenon in natural products. Finally, some common potential hypertension biomarkers for all test compounds were discovered. These biomarkers can be used as an endogenous efficacy index for clinically evaluating the antihypertensive effect of the medicinal substances from natural products.

## Data Availability Statement

All datasets generated for this study are included in the article/**Supplementary Material**.

## Ethics Statement

The animal study was reviewed and approved by the animal protection and use committee of Shandong University of Traditional Chinese Medicine.

## Author Contributions

ZT and SZ contributed to the study design, study conduct, and drafting of the manuscript. HW, ZC, and MS contributed to the data collection, data interpretation. LS and LG contributed to data analysis. HJ and YL revised the manuscript.

## Funding

This study was supported by the foundation from the National Natural Science Foundation of China (no. 81473653, and no. 81774173), Major Science and Technology Innovation Project in Shandong Province (No. 2017CXGC1307), Key Research and Development project of Shandong Province (2018GSF119007).

## Conflict of Interest

The authors declare that the research was conducted in the absence of any commercial or financial relationships that could be construed as a potential conflict of interest.

## References

[B1] Aoki J. Taira A. Takanezawa Y. Kishi Y. Hama K. Kishimoto T. (2002). Serum lysophosphatidic acid is produced through diverse phospholipase pathways. J. Biol. Chem. 277, 48737–48744. 10.1074/jbc.M206812200 12354767

[B2] Bacon S. L. Sherwood A. Hinderliter A. Blumenthal J. A. (2004). Effects of exercise, diet and weight loss on high blood pressure. Sports Med. 34, 7–16. 10.2165/00007256-200434050-00003 15107009

[B3] Biernacki M. Ambrożewicz E. Gęgotek A. Toczek M. Skrzydlewska E. (2019). Long-term administration of fatty acid amide hydrolase inhibitor (URB597) to rats with spontaneous hypertension disturbs liver redox balance and phospholipid metabolism. Adv. Med. Sci. 64, 15–23. 10.1016/j.advms.2018.06.002 30243113

[B4] Bourbon N. A. Sandirasegarane L. Kester M. (2002). Ceramide-induced inhibition of Akt is mediated through protein kinase Czeta: implications for growth arrest. J. Biol. Chem. 277, 3286–3292. 10.1074/jbc.M110541200 11723139

[B5] Cardiff R. D. Miller C. H. Munn R. J. (2014). Manual hematoxylin and eosin staining of mouse tissue sections. Cold Spring Harb. Protoc. 2, 655–658. 10.1101/pdb.prot073411 24890205

[B6] Chen H. Chen L. Liu D. Chen D. Q. Vaziri N. D. Yu X. Y. (2017). Combined clinical phenotype and lipidomic analysis reveals the impact of chronic kidney disease on lipid metabolism. J. Proteome Res., 16, 1566–1578. 10.1021/acs.jproteome.6b00956 28286957

[B7] Davis V. W. Schiller D. E. Eurich D. Sawyer M. B. (2012). Urinary metabolomic signature of esophageal cancer and Barrett's esophagus. World J. Surg. Oncol. 10, 271. 10.1186/1477-7819-10-271 23241138 PMC3579706

[B8] Do G. M. Jung U. J. Park H. J. Kwon E. Y. Jeon S. M. McGregor R. A. (2012). Resveratrol ameliorates diabetes-related metabolic changes via activation of AMP-activated protein kinase and its downstream targets in db/db mice. Mol. Nutr. Food Res. 56, 1282–1291. 10.1002/mnfr.201200067 22715031

[B9] Dorrance A. M. Graham D. Webb R. C. Fraser R. Dominiczak A. (2001). Increased membrane sphingomyelin and arachidonic acid in stroke-prone spontaneously hypertensive rats. Am. J. Hypertens. 14, 1149–1153. 10.1016/s0895-7061(01)02188-4 11724215

[B10] Feng Z. J. Hou J. J. Yu Y. Wu W. Y. Deng Y. P. Wang X. (2019). Dissecting the metabolic phenotype of the antihypertensive effects of five Uncaria species on spontaneously hypertensive rats. Front. Pharmacol. 10, 845. 10.3389/fphar.2019.00845 31417403 PMC6682664

[B11] Graessler J. Schwudke D. Schwarz P. E. H. Herzog R. Shevchenko A. Bornstein S. R. (2009). Top-down lipidomics reveals ether lipid deficiency in blood plasma of hypertensive patients. PloS One 4, e6261. 10.1371/journal.pone.0006261 19603071 PMC2705678

[B12] Guo S. X. Yan Y. Z. Mu L. T. Niu Q. He J. Liu J. M. (2015). Association of serum free fatty acids with hypertension and insulin resistance among rural Uyghur adults in far western China. Int. J. Environ. Res. Public Health 12, 6582–6590. 10.3390/ijerph120606582 26067991 PMC4483717

[B13] Hao H. F. Liu L. M. Pan C. S. Wang C. S. Gao Y. S. Fan J. Y. (2017). Rhynchophylline ameliorates endothelial dysfunction via Src-PI3K/Akt-eNOS cascade in the cultured intrarenal arteries of spontaneous hypertensive rats. Front. Physiol. 8, 928. 10.3389/fphys.2017.00928 29187825 PMC5694770

[B14] Heitzman M. E. Neto C. C. Winiarz E. Vaisberg A. J. Hammond G. B. (2005). Ethnobotany, phytochemistry and pharmacology of *Uncaria* (Rubiaceae). Phytochemistry 66, 5–29. 10.1016/j.phytochem.2004.10.022 15649507

[B15] Hinterwirth H. Stegemann C. Mayr M. (2014). Lipidomics: quest for molecular lipid biomarkers in cardiovascular disease. Circ. Cardiovasc. Genet. 7, 941–954. 10.1161/CIRCGENETICS.114.000550 25516624

[B16] Hirayama T. Ogawa Y. Tobise K. Kikuchi K. (1998). Mechanism of endothelium-dependent vasorelaxation evoked by lysophosphatidylcholine. Hypertens. Res. 21, 137–145. 10.1291/hypres.21.137 9786596

[B17] Hu C. Hoene M. Zhao X. Häring H. U. Schleicher E. Lehmann R. (2010). Lipidomics analysis reveals efficient storage of hepatic triacylglycerides enriched in unsaturated fatty acids after one bout of exercise in mice. PloS One 5, e13318. 10.1371/journal.pone.0013318 20967198 PMC2954156

[B18] Hu C. X. Kong H. W. Qu F. X. Li Y. Yu Z. Q. Gao P. (2011). Application of plasma lipidomics in studying the response of patients with essential hypertension to antihypertensive drug therapy. Mol. Biosyst. 7, 3271–3279. 10.1039/c1mb05342f 22009255

[B19] Iverson S. J. Lang S. L. Cooper M. H. (2001). Comparison of the Bligh and Dyer and Folch methods for total lipid determination in a broad range of marine tissue. Lipids 36, 1283–1237. 10.1007/s11745-001-0843-0 11795862

[B20] Kulkarni H. Meikle P. J. Mamtani M. Weir J. M. Barlow C. K. Jowett J. B. (2013). Plasma lipidomic profile signature of hypertension in Mexican American families: specific role of diacylglycerols. Hypertension 62, 621–626. 10.1161/HYPERTENSIONAHA.113.01396 23798346 PMC3789127

[B21] Kwan H. Y. Hu Y. M. Chan C. L. Cao H. H. Cheng C. Y. Pan S. Y. (2013). Lipidomics identification of metabolic biomarkers in chemically induced hypertriglyceridemic mice. J. Proteome Res. 12, 1387–1398. 10.1021/pr3010327 23336740

[B22] Kwong E. Li Y. Hylemon P. B. Zhou H. (2015). Bile acids and sphingosine-1-phosphate receptor 2 in hepatic lipid metabolism. Acta Pharm. Sin. B. 5, 151–157. 10.1016/j.apsb.2014.12.009 26579441 PMC4629213

[B23] Lam S. M. Wang Z. H. Li J. Huang X. Shuai G. H. (2017). Sequestration of polyunsaturated fatty acids in membrane phospholipids of Caenorhabditis elegans dauer larva attenuates eicosanoid biosynthesis for prolonged survival. Redox Biol. 12, 967–977. 10.1016/j.redox.2017.05.002 28499251 PMC5429230

[B24] Law S. H. Chan M. L. Marathe G. K. Parveen F. Chen C. H. Ke L. Y. (2019). An updated review of lysophosphatidylcholine metabolism in human diseases. Int. J. Mol. Sci. 20, 1149. 10.3390/ijms20051149 PMC6429061 30845751

[B25] Li P. Y. Zeng X. R. Cheng J. Wen J. Inoue I. Yang Y. (2013). Rhynchophylline-induced vasodilation in human mesenteric artery is mainly due to blockage of L-type calcium channels in vascular smooth muscle cells. N-S Arch. Pharmacol. 386, 973–982. 10.1007/s00210-013-0888-6 23812676

[B26] Liu A. N. Chu Y. J. Wang X. M. Yu R. X. Jiang H. Q. Li Y. L. (2018). Serum metabolomics study based on LC-MS and antihypertensive Effect of *Uncaria* on spontaneously hypertensive rats. Evid. Based. Compl. Alt., 2018, 9281946. 10.1155/2018/9281946 PMC5904782 29849735

[B27] Ma N. Yang Y. J. Liu X. W. Kong X. J. Li S. H. Qin Z. (2017). UPLC-Q-TOF/MS-based metabonomic studies on the intervention effects of aspirin eugenol ester in atherosclerosis hamsters. Sci. Rep. 7, 10544. 10.1038/s41598-017-11422-7 28874840 PMC5585262

[B28] Marteau J. B. Zaiou M. Siest G. Visvikis-Siest S. (2005). Genetic determinants of blood pressure regulation. J. Hypertens. 23, 2127–2143. 10.1097/01.hjh.0000186024.12364.2e 16269952

[B29] Matsutomo T. Ushijima M. Kodera Y. Nakamoto M. Takashima M. Morihara N. (2017). Metabolomic study on the antihypertensive effect of S-1-propenylcysteine in spontaneously hypertensive rats using liquid chromatography coupled with quadrupole-Orbitrap mass spectrometry. J. Chromatogr. B. Analyt. Technol. Biomed. Life Sci. 1046, 147–155. 10.1016/j.jchromb.2017.01.029 28183044

[B30] Miao H. Chen H. Pei S. Bai X. Vaziri N. D. Zhao Y. Y. (2015). Plasma lipidomics reveal profound perturbation of glycerophospholipids, fatty acids, and sphingolipids in diet-induced hyperlipidemia. Chem. Biol. Interact. 228, 79–87. 10.1016/j.cbi.2015.01.023 25619641

[B31] Miao H. Zhao Y. H. Vaziri N. D. Tang D. D. Chen H. Chen H. (2016). Lipidomics biomarkers of diet-induced hyperlipidemia and its treatment with *Poria cocos* . J. Agric. Food Chem. 64, 969–979. 10.1021/acs.jafc.5b05350 26758241

[B32] Milne S. Ivanova P. Forrester J. Alex B. H. (2006). Lipidomics: an analysis of cellular lipids by ESI-MS. Methods 39, 92–103. 10.1016/j.ymeth.2006.05.014 16846739

[B33] Mori T. A. (2006). Omega-3 fatty acids and hypertension in humans. Clin. Exp. Pharmacol. Physiol. 33, 842–846. 10.1111/j.1440-1681.2006.04451.x 16922818

[B34] Mulders A. C. M. Hendriks-Balk M. C. Mathy M. J. Michel M. C. Alewijnse A. E. Peters S. L. M. (2006). Sphingosine kinase–dependent activation of endothelial nitric oxide synthase by angiotensin II. Arterioscler. Thromb. Vasc. Biol. 26, 2043–2048. 10.1161/01.ATV.0000237569.95046.b9 16857953

[B35] Mulders A. C. M. Mathy M. J. Zu Heringdorf D. M. ter Braak M. Hajji N. Olthof D. C. (2009). Activation of sphingosine kinase by muscarinic receptors enhances NO-mediated and attenuates EDHF-mediated vasorelaxation. Basic Res. Cardiol. 104, 50–59. 10.1007/s00395-008-0744-x 18777003

[B36] Ndagijimana A. Wang X. Pan G. Zhang F. Feng H. Olaleye O. (2013). A review on indole alkaloids isolated from Uncaria rhynchophylla and their pharmacological studies. Fitoterapia 86, 35–47. 10.1016/j.fitote.2013.01.018 23376412

[B37] Nguyen P. Leray V. Diez M. Serisier S. Le, Bloc'h J. Siliart B. (2008). Liver lipid metabolism. J. Anim. Physiol. Anim. Nutr. (Berl) 92, 272–283. 10.1111/j.1439-0396.2007.00752.x 18477307

[B38] Qin Z. Wang W. Liao D. Wu X. Li X. (2018). UPLC-Q/TOF-MS-based serum metabolomics reveals hypoglycemic effects of rehmannia glutinosa, coptis chinensis and their combination on high-fat-diet-induced diabetes in KK-Ay mice. Int. J. Mol. Sci. 19, E3984. 10.3390/ijms19123984 30544908 PMC6320869

[B39] Rubingh C. M. Bijlsma S. Derks E. P. Bobeldijk I. Verheij E. R. Kochhar S. (2006). Assessing the performance of statistical validation tools for megavariate metabolomics data. Metabolomics 2, 53–61. 10.1007/s11306-006-0022-6 24489531 PMC3906710

[B40] Shi J. Yu J. Chen X. Xu R. (2003). Pharmacological actions of Uncaria alkaloids, rhynchophylline and isorhynchophylline. Acta Pharmacol. Sin. 24, 97–101. 10.1021/ar020052o 12546715

[B41] Spijkers L. J. A. van den Akker R. F. P. Janssen B. J. A. Debets J. J. De Mey J. G. R. Stroes E. S. G. (2011). Hypertension is associated with marked alterations in sphingolipid biology: a potential role for ceramide. PloS One 6, e21817. 10.1371/journal.pone.0021817 21818267 PMC3139577

[B42] Tian Y. P. Jiang F. Li Y. L. Jiang H. Q. Chu Y. J. Zhu L. J. (2018). Evaluation of the anti-hypertensive effect of Tengfu Jiangya tablet by combination of UPLC-Q-exactive-MS-based metabolomics and iTRAQ-based proteomics technology. Biomed. Pharmacother. 100, 324–334. 10.1016/j.biopha.2018.02.025 29453042

[B43] Ubhi B. K. (2018). Direct infusion-tandem mass spectrometry (DI-MS/MS) analysis of complex lipids in Human plasma and serum using the Lipidyzer™ Platform. Methods Mol. Biol. 1730, 227–236. 10.1007/978-1-4939-7592-1_15 29363076

[B44] Urbina E. Alpert B. Flynn J. Hayman L. Harshfield G. A. Jacobson M. (2008). Ambulatory blood pressure monitoring in children and adolescents: recommendations for standard assessment: a scientific statement from the American Heart Association Atherosclerosis, Hypertension, and Obesity in Youth Committee of the council on cardiovas. Hypertension 52, 433–451. 10.1016/s0145-4145(08)79423-1 18678786

[B45] Walther A. Cannistraci C. V. Simons K. Gerl M. J. Duran C. Wehrli S. (2018). Lipidomics in major depressive disorder. Front. Psychiatry 9, 459. 10.3389/fpsyt.2018.00459 30374314 PMC6196281

[B46] Wolf A. Saito T. Dudek R. Bing R. J. (1991). The effect of lysophosphatidylcholine on coronary and renal circulation in the rabbit. Lipids 26, 223–226. 10.1007/bf02543975 2046490

[B47] Xie J. Jiang H. Q. Li Y. L. Nie L. Zhou H. L. Yang W. Q. (2019). Study on the intervention effects of Pinggan Prescription (平肝方) on spontaneously hypertensive rats based on metabonomic and pharmacodynamic methods. Chin. J. Integr. Med. 25, 348–353. 10.1007/s11655-015-2126-1 28028715

[B48] Zhang W. B. Chen C. X. Sim S. M. Kwan C. Y. (2004). In vitro vasodilator mechanisms of the indole alkaloids rhynchophylline and isorhynchophylline, isolated from the hook of Uncaria rhynchophylla (Miquel). N-S Arch. Pharmacol. 369, 232–238. 10.1007/s00210-003-0854-9 14668978

[B49] Zhang F. Sun A. S. Yu L. M. Wu Q. Gong Q. H. (2008). Effects of isorhynchophylline on angiotensin II-induced proliferation in rat vascular smooth muscle cells. J. Pharm. Pharmacol. 60, 1673–1678. 10.1211/jpp/60.12.0014 19000373

[B50] Zhang S. M. Qi D. M. Cao Y. M. Zhou H. L. Jiang H. Q. Li Y. L. (2019). Lipidomics study on intervention by Uncaria on hepatic metabolic disorder in spontaneously hypertensive rats. Acta Pharm. Sinica 54, 1636–1644. 10.16438/j.0513-4870.2019-0164

[B51] Zhou J. Zhou S. (2010). Antihypertensive and neuroprotective activities of rhynchophylline: the role of rhynchophylline in neurotransmission and ion channel activity. J. Ethnopharmacol. 132, 15–27. 10.1016/j.jep.2010.08.041 20736055

[B52] Zhou J. Y. Zhou S. W. (2012). Isorhynchophylline: A plant alkaloid with therapeutic potential for cardiovascular and central nervous system diseases. Fitoterapia 83, 617–626. 10.1016/j.fitote.2012.02.010 22406453

[B53] Zhu Y. Liu H. Zhang M. Guo G. L. (2016). Fatty liver diseases, bile acids, and FXR. Acta Pharm. Sin. B. 6, 409–412. 10.1016/j.apsb.2016.07.008 27709009 PMC5045552

[B54] Zhuang X. Deng Z. B. Mu J. Zhang L. Yan J. Miller D. (2015). Ginger-derived nanoparticles protect against alcohol-induced liver damage. J. Extracell Vesicles 4, 8713. 10.3402/jev.v4.28713 PMC4662062 26610593

