# Supplementary material for: Intervention of Uncaria and Its Components on Liver Lipid Metabolism in Spontaneously Hypertensive Rats
Source: Front Pharmacol. 2020 Jul 16;11:910. doi: 10.3389/fphar.2020.00910 (PMC7381107; doi:10.3389/fphar.2020.00910)
Supplement: Supplementary file 1 [file DataSheet_1.docx]

Supplementary Material

# Supplementary Data

**Materials and Methods**

**Materials**

*Uncaria rhynchophylla* (Miq.) Miq. ex Havil (origin from Hunan Province ) was identified by Professor Xu Lingchuan, Shandong University of Traditional Chinese Medicine. Rhynchophylline (ST07840120MG-4894), dehydrorhynchophylline (ST10570120MG-2916) and isodehydrorhynchophylline (ST11080120MG-2917) were purchased from Shanghai Standard Technology Co., Ltd., isorhynchophylline (1228A021) from Beijing Solarbio Science & Technology Co., Ltd., diphenylguanidine (internal standard (IS))(Z03N8Y47297) from Shanghai Yuanye Bio-Technology Co., Ltd. Acetonitrile, methanol and formic acid used were gradient grade for liquid chromatography supplied from Thermo Fisher Company (USA). Ammonium Formate used was chromatographic grade from Haimai Kun Chemical Company (USA).

**Standard solutions preparation**

The reference substances of rhynchophylline, isorhynchophylline, dehydrorhynchophylline and isodehydrorhynchophylline were precisely weighed and added with methanol to yield concentration of 52.2 μg·mL^-1^, 50 μg·mL^-1^, 46.8 μg·mL^-1^ and 55.6 μg·mL^-1^ respectively. The standard product of diphenylguanidine, which was used as the internal standard (IS), was weighed and volume was fixed with methanol to obtain concentration of 1.101 μg·mL^-1^.

**Sample preparation**

The dried whole plant, *Uncaria rhynchophylla* (Miq.) Miq. ex Havil ( No. 170906) , was purchased from Huqiao Pharmaceutical Co., Ltd. (Bozhou, Anhui, China). The plant was authenticated by Professor Feng Li, School of Pharmacy, Shandong University of Traditional Chinese Medicine, Shandong. The leaves were removed from the whole plant and the remaining plant (stem & root) were weighed, crushed and extracted with 100 mL 95% ethanol of at room temperature for 3 times, 1 hour each time. The residue was extracted 3 times with 10 times volume of water by reflux, 1 hour each time, and then centrifugally filtered. The ethanol and water extracts were recovered under pressure and concentrated to obtain a concentration of 1 μg·mL^-1^. The two solutions were then combined. 0.5 mL of extract was added with 9.5 mL of methanol containing 1 μg·mL^-1^ IS and then ultrasounded for 45 minutes. The extract was filtered and the filtrate obtained was used as the stock solution for further quantitative analysis of alkaloids.

**UPLC analysis**

The UPLC/MS system used consists of a UltiMate 3000 Ultra High Performance Liquid Chromatography (Thermo Fisher Scientific, USA ) connected to an Quadrupole-Electrostatic (Q Exactive) field orbital trap high resolution mass spectrometry (Thermo Fisher Scientific, USA) and is equipped with ESI ion source. UPLC was performed using a 2.7 μm, 100 ×2.1 mm，Halo C_18_ column (Advanced Materials Technology, USA) operated in gradient mode at 0.3 mL·min^-1^ with a run time of 8 minutes. Solvent A was water and solvent B was acetonitrile containing 0.05% formic acid. The mobile phase composition are as shown in **Supplementary Table 1**. The MS was operated in electrospray positive ionisation mode. The instrument settings were as follows: source temperature of 350 ℃; curtain gas of 45; Ion Spray voltage of 3,500 V. S-Lens RF Level was set at 55 and fragmentation energy values of MS/MS secondary mass spectrometry analysis were 20, 50 and 65. Data collected were analyzed using Xcalibur software (Thermo Fisher Scientific, USA).

**MS analysis**

The total ion chromatograms (TIC) and mass spectra of Rhynchophylline, Isorhynchophylline, Corynoxeine and Isocorynoxeine are as shown in **Supplementary** **Figure 1**.

Rhynchophylline and isorhynchophylline are isomers with relative molecular weight of 384.4. Due to the action of formic acid in mobile phase, they ionise to form [M+H]^+^ ions with molecular weight of 385.211. The retention time of isorhynchophylline and rhynchophylline are 5.56 minutes and 6.13 minutes respectively (**Supplementary** **Figure 1B**). Dehydrorhynchophylline and isodehydrorhynchophylline are enantiomers with molecular weight of 382.45. They ionise to form give [M+H]^+^ peaks with molecular weight of 383.196. The retention time of isodehydrorhynchophylline and dehydrorhynchophyllline are 5.31 minutes and 5.68 minutes respectively (**Supplementary** **Figure 1C**).

**Method validation**

The LC-MS/MS method was validated for linearity, precision, reproducibility, stability and recovery according to published guidance for method.

Linearity study: Diphenylguanidine solutions were used as the starting calibrator solution. Mixed reference substances were injected continuously from low to high concentration accordingly (**Supplementary Table 2**), with 2 uL at a time, and this step was repeated twice. The limit of detection (LOD) was 3 times of signal-to-noise ratio and the limit of quantitative (LOQ) was 10 times of signal-to-noise ratio. The average peak area ratio of standard products and diphenylguanidine were determined twice. Linear regression equation was calculated by taking the concentration of rhynchophylline, isorhynchophylline, dehydrorhynchophylline and isodehydrorhynchophylline as X-axis and the ratio of peak area of standard product to diphenylguanidine of internal standard as Y-axis.

According to the method above, the interassay precision of the four components was investigated using the stock solution for 6 consecutive injections. 3 stock solutions were used to determine the reproducibility. The stability of the stock solution at 0th h, 2nd h, 4th h, 6th h, 12th h and 16th h was investigated. RSD values of four components were calculated using peak area as index. The stock solution was divided into three parts, and the control substances of high, middle and low concentrations were added into the solution respectively. The average recovery of each sample was determined three times.

**Content determination**

Three replicateswere taken for the stock solution, and the content of four alkaloids were determined. The content of the alkaloids was expressed in the form of $\overline{\text{X}}$±SD.

**RESULTS**

**Method validation**

The aim of this study was to develop and validate a method for quantification of four main alkaloids of *Uncaria*. During validation of method allowed determination of rhynchophylline from 4.52 μg·mL to 9040 μg·mL and isorhynchophylline up to 9920 μg·mL (**Supplementary Table 3**). RSD of the four alkaloids ranged from 1.28% to 4.06%. The results obtained are as shown in Table 3, which shows that the instrument used had a good precision. The results of stability and repeatability are shown in **Supplementary Table 4**, indicating that this system was stable, and the experiments were repeatable using this system. The average recovery of rhynchophylline, isorhynchophylline, dehydroisorhynchophylline and isodehydrorhynchophylline was 102.0%, 102.6%, 105.1% and 102.7% respectively.

**Content**

The content of four alkaloids determined are as shown in **Supplementary Table 9**. As shown in Table 8, the content of rhynchophylline and isorhynchophylline are much higher than dehydrorhynchophylline and isodehydrorhynchophylline.

# UPLC-Q/Orbitrap-MS method validation

The total ion chromatograms of the fatty acid species and lipid species from WKY, SHR, UET RT and IT are shown in Supplementary Figure 1. To further illustrate the capability of the reversed-phase UPLC/MS system for the analysis of fatty acids and lipids, these fatty acids and lipids were extracted from rat liver and analysed with the same method parameters. Both positive and negative ion electrosprays were utilised because not all lipid species may ionise effectively in one mode.

The liver samples were analyzed within 29 minutes by UPLC-Q/Orbitrap -MS in both positive and negative ESI detection modes. The representative chromatograms are shown in Figure S5. To guarantee the significant differences of liver metabolites in LC-MS originating from the inherent differences between groups rather than from the instrumental drift, the instrument stability and analytical repeatability were evaluated by analysing the quality control (QC) samples during the analytical run. The instrument repeatability and method repeatability were validated by analysing one QC sample for six times continuously to obtain six replicates. For the instrument and method repeatability, ten ion peaks of the fatty acid species in negative ion mode, lipid species in positive ions mode and lipid species in negative ions mode were extracted for method validation. For instrument repeatability, relative standard deviation (RSD)% of the peak intensities for the fatty acid species in negative ion mode, lipid species in positive ions mode and lipid species in negative ions mode were estimated to be 1.42–3.79%, 0.91–5.08 % and 2.67–8.05 %, respectively; for the method repeatability, their RSD% values of the peak intensities for the fatty acid species in negative ion mode, lipid species in positive ions mode and lipid species in negative ions mode were 1.34–7.25%, 0.72-2.75 % and 2.11–9.18%.as shown in **Supplementary Tables 10, 11** and **12**, respectively. Meanwhile, the deviation variation of all QC samples was further accessed via principal component analysis for method validation. The results obtained show that deviation of the 12 QC samples in both positive ion mode and six in negative ion mode fell within 2×SD and 95% confidence interval (**Supplementary** **Figure 2**). QC samples were also further subjected to principal component analysis (PCA) together with the experimental samples (**Supplementary** **Figure 3**). According to the score plots, most QC samples were clustered closely, indicating that the analytical platform provided an excellent precision and repeatability required for a large-scale metabolomics study.

# Identification of fatty acids

The identification of fatty acids was carried out in the electrospray negative ion mode. The data fragments were matched with the standard data. According to the results as shown in **Supplementary** **Figure 4**, the sample data matched the standard data perfectly, implying that the material identification was accurate. The peak value of molecular ions was [M-H]-, which produced fragment ions [-(CH_2_) _3_COOH]^-^ with m/z value of 87.00714.

# Supplementary Figures and Tables

## Supplementary Figures


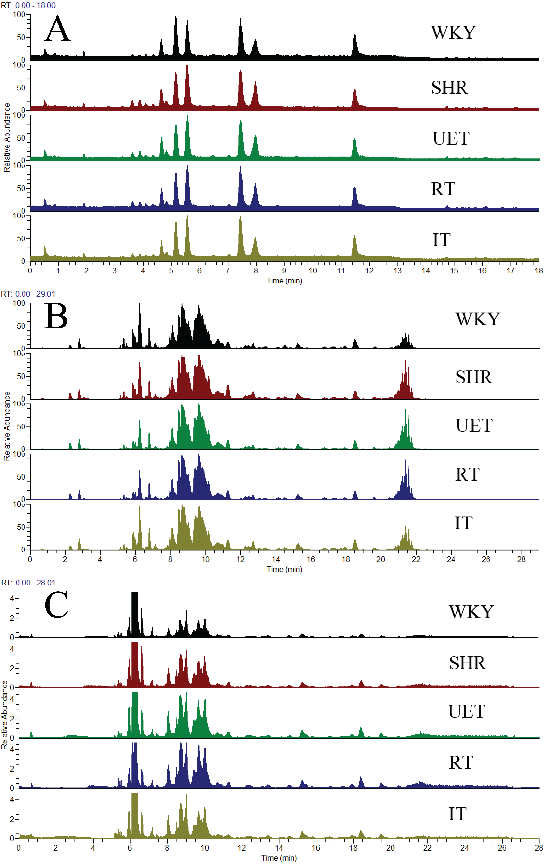


**Supplementary** **Figure 1.** Total ion current (TIC) chromatogram of UPLC/MS separation of analytes extracted from the supernatant of protein precipitated in rat liver; **(A)** Fatty acid species in negative ions mode, **(B)** Lipid species in positive ions mode and **(C)** Lipid species in negative ions mode.


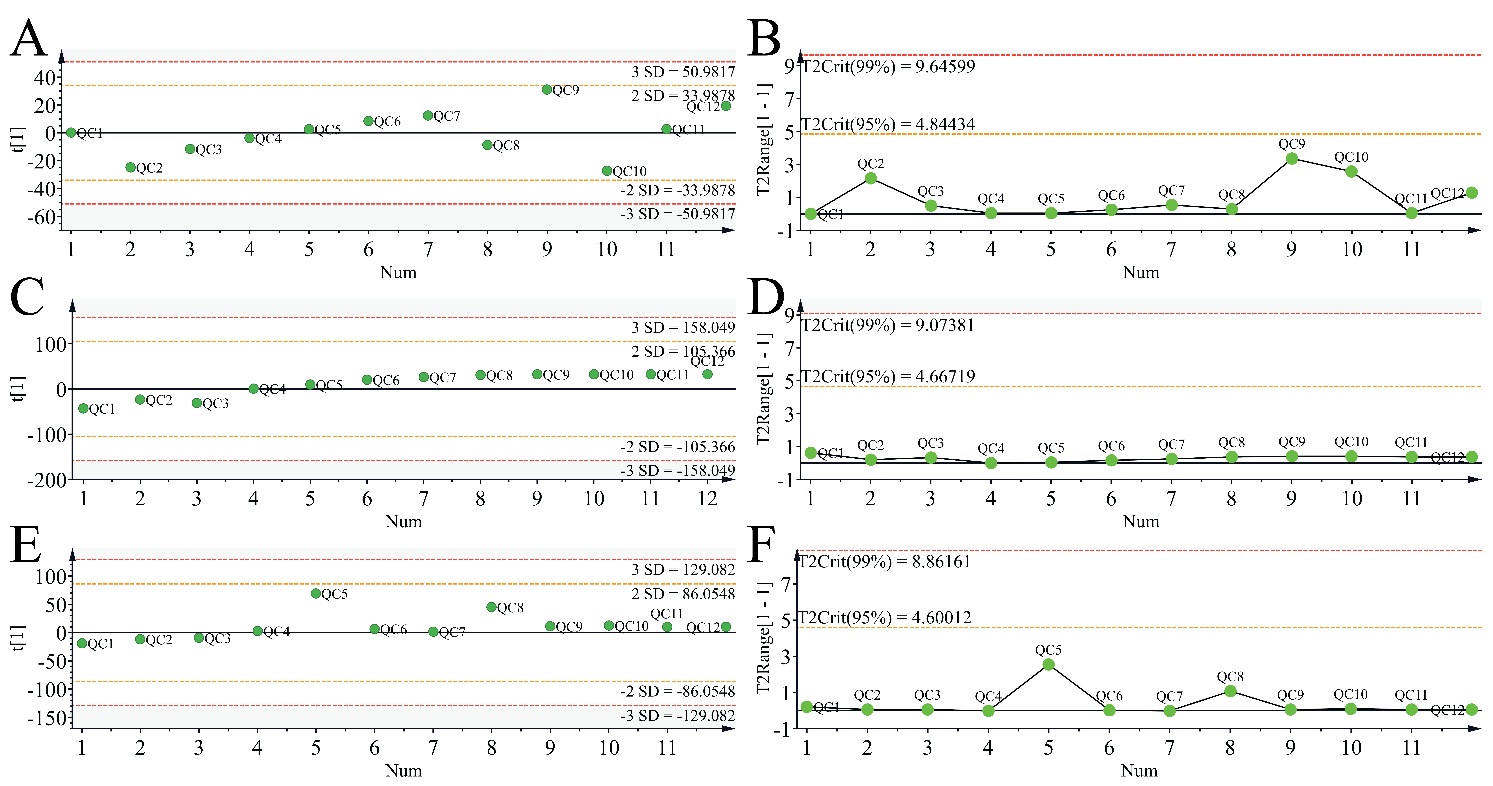


**Supplementary** **Figure 2.** PCA line score plots of different injections of quality control (QC) sample. X-axis represents the run order of QC sample; Y-axis represents standard deviation (**A**, **C**, **E**) and Hotelling’s T2 range (**B**, **D**, **F**). (**A**, **B**) represents PCA line score plots for fatty acid species in negative ion mode, (**C**,**D**) represents PCA line score plots for lipid species in positive ion mode and (**E**,**F**) represents lipid species in positive ion mode.


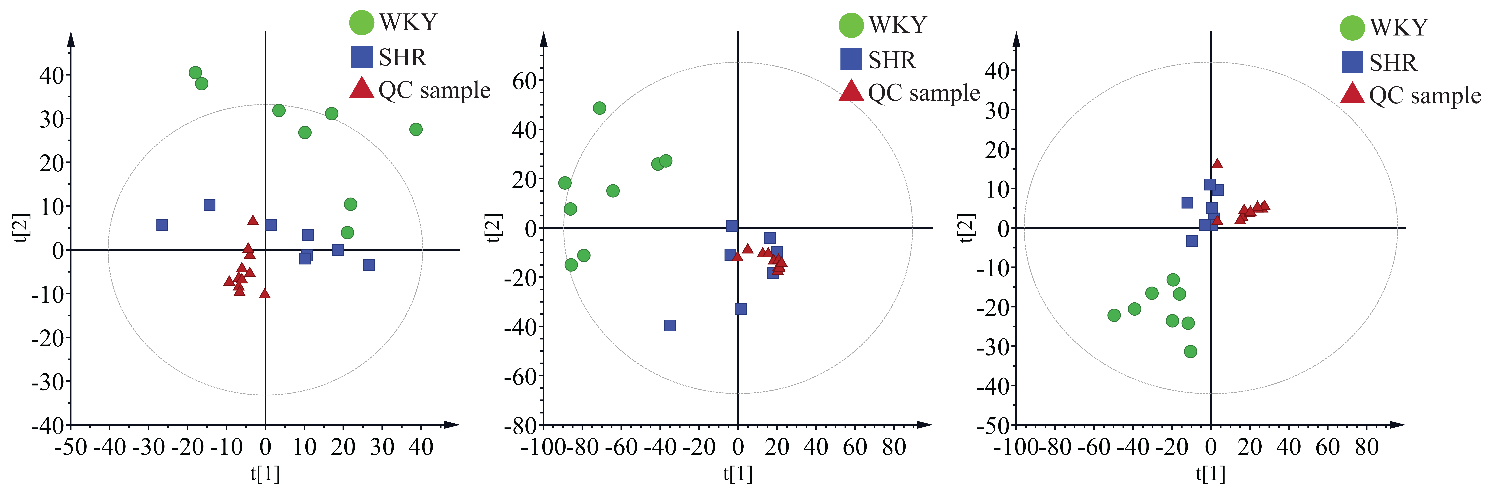


**Supplementary** **Figure 3**. PCA score plots derived from UPLC-Q-Orbitrap-MS profiling of fatty acid and lipid species of WKY group, SHR groups, and QC sample. **(A)** represents PCA score plots for fatty acid species in negative ions mode, **(B)** represents lipid species in positive ions mode and **(C)** represents lipid species in negative ions mode.


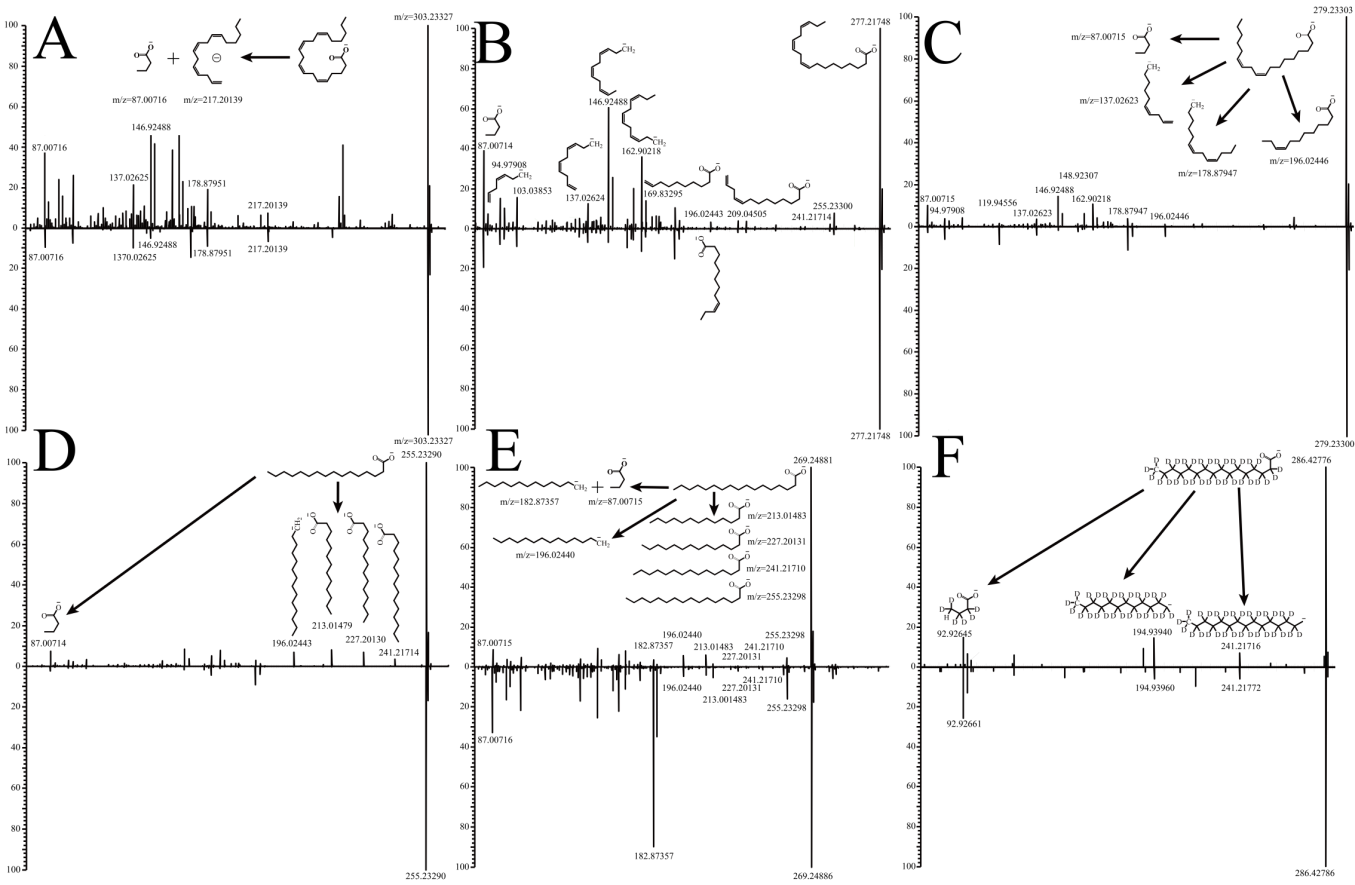


**Supplementary** **Figure 4.** Mass spectrometry analysis of potential biomarkers of fatty acids. **(A)** represents mass spectrometry analysis for Arachidonic acid (m/z 303.23327), **(B)** represents mass spectrometry analysis for α-linolenic acid (m/z 277.21748), **(C)** represents mass spectrometry analysis for linoleic acid (m/z 279.23303), **(D)** represents mass spectrometry analysis for palmitic acid (m/z 255.23290), **(E)** represents mass spectrometry analysis for heptadecanoic acid (m/z 269.24886), **(F)** represents mass spectrometry analysis for d31-palmitic acid (IS) (m/z 286.42786). The data of sample fragments are above the coordinate axis, and the data of standard fragments are below the coordinate axis.


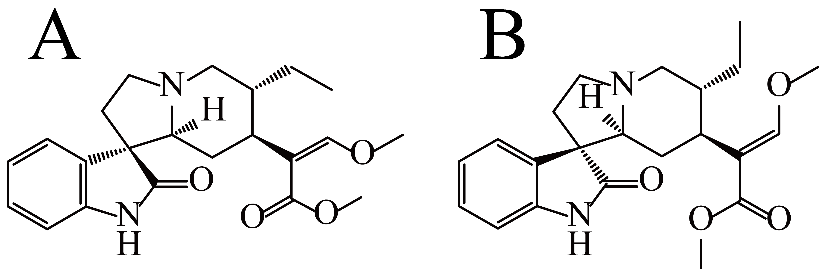


**Supplementary** **Figure 5.** Chemical structures of rhynchophylline **(A)** and isorhynchophylline **(B)**.

## Supplementary Tables

## Supplementary Table 1. Mobile phase composition for UPLC.

| **Time** (min) | **A** (%) | **B** (%) |
| --- | --- | --- |
| 0 | 95 | 5 |
| 3 | 80 | 20 |
| 8 | 70 | 30 |

**Supplementary Tables 2.** Concentration of four alkaloids in *Uncaria*.

| **Rhynchophylline** (ng·mL^-1^) | **Isorhynchophylline** (ng·mL^-1^) | **Dehydrorhynchophylline** (ng·mL^-1^) | **Isodehydrorhynchophylline** (ng·mL^-1^) |
| --- | --- | --- | --- |
| 10440 | 10000 | 11120 | 9360 |
| 2088 | 2000 | 2780 | 2340 |
| 417.6 | 500.0 | 556.0 | 585.0 |
| 83.52 | 100.0 | 111.2 | 117.0 |
| 16.70 | 20.00 | 22.24 | 23.40 |
| 3.341 | 4.000 | 4.448 | 4.680 |

**Supplementary Tables 3.** Standard curve, R^2^, linear range, LOQ and LOD of components in *Uncaria*.

| **Analyst** | **Standard curve** | **R^2^** | **Linear range**  (μg·mL^-1^) | **LOQ** (μg·mL^-1^) | **LOD**  (μg·mL^-1^) |
| --- | --- | --- | --- | --- | --- |
| Rhynchophylline | y=1.2163*10^-2^+2.20715*10^-4^x | 0.9986 | 4.52-9040 | 0.348 | 0.104 |
| Isorhynchophylline | y=-7.0136*10^-5^+2.4241*10^-4^x | 1.0000 | 4.96-9920 | 0.300 | 0.100 |
| Dehydrorhynchophylline | y=4.9287*10^-4^+2.4241*10^-4^x | 0.9932 | 5.48-10960 | 0.312 | 0.094 |
| Isodehydrorhynchophylline | y=2.0310*10^-2^+1.92655*10^-4^x | 0.9960 | 4.90-9800 | 0.370 | 0.111 |

**Supplementary Tables 4.** Results of stability, repeatability and precision.

| **Analyst** | **Stability RSD**  **(**%) | **Repeatability RSD**  (%) | **Precision RSD**  (%) |
| --- | --- | --- | --- |
| Rhynchophylline | 2.28 | 3.69 | 0.78 |
| Isorhynchophylline | 1.97 | 1.20 | 1.59 |
| Dehydrorhynchophylline | 1.10 | 1.97 | 1.21 |
| Isodehydrorhynchophylline | 2.75 | 3.13 | 1.18 |

**Supplementary Tables 5.** Recovery of rhynchophylline

| **No.** | **Content**  (μg·mL^-1^) | **Addition of rhynchophylline**  (μg·mL^-1^) | **Measured content**  (μg·mL^-1^) | **Recovery**  (%) | **Average recovery**  (%) |
| --- | --- | --- | --- | --- | --- |
| 1 | 10859.8 | 8000.0 | 18262.1 | 92.6 | 102.0 |
| 2 | 10859.8 | 8000.0 | 18980.9 | 101.6 |
| 3 | 10859.8 | 8000.0 | 18448.6 | 94.9 |
| 4 | 10972.1 | 500.0 | 11528.9 | 111.4 |
| 5 | 10972.1 | 500.0 | 11409.4 | 82.1 |
| 6 | 10972.1 | 500.0 | 11591.9 | 116.3 |
| 7 | 10873.5 | 100.0 | 10991.3 | 117.9 |
| 8 | 10873.5 | 100.0 | 10973.0 | 99.6 |
| 9 | 10873.5 | 100.0 | 10974.9 | 101.4 |

**Supplementary Tables 6.** Recovery of isorhynchophylline.

| **No**. | **Content**  (μg·mL^-1^) | **Addition of isorhynchophylline**  (μg·mL^-1^) | **Measured content**  (μg·mL^-1^) | **Recovery**  (%) | **Average recovery**  (%) |
| --- | --- | --- | --- | --- | --- |
| 1 | 6414.7 | 5900.0 | 12072.5 | 97.2 | 102.6 |
| 2 | 6414.7 | 5900.0 | 12133.5 | 98.3 |
| 3 | 6414.7 | 5900.0 | 12154.6 | 98.6 |
| 4 | 6162.4 | 590.0 | 6793.2 | 106.9 |
| 5 | 6162.4 | 590.0 | 6828.3 | 112.9 |
| 6 | 6162.4 | 590.0 | 6748.6 | 99.4 |
| 7 | 6326.6 | 196.7 | 6552.7 | 114.9 |
| 8 | 6326.6 | 196.7 | 6521.3 | 99.0 |
| 9 | 6326.6 | 196.7 | 6515.9 | 96.2 |

**Supplementary Tables 7.** Recovery of dehydrorhynchophylline.

| **No.** | **Content**  (μg·mL^-1^) | **Addition of dehydrorhynchophylline**  (μg·mL^-1^) | **Measured content**  (μg·mL^-1^) | **Recovery**  (%) | **Average recovery**  (%) |
| --- | --- | --- | --- | --- | --- |
| 1 | 2813.3 | 4200.0 | 6934.8 | 98.1 | 105.1 |
| 2 | 2813.3 | 4200.0 | 7196.3 | 104.4 |
| 3 | 2813.3 | 4200.0 | 7239.2 | 105.4 |
| 4 | 2749.3 | 280.0 | 3048.5 | 106.9 |
| 5 | 2749.3 | 280.0 | 2957.1 | 74.2 |
| 6 | 2749.3 | 280.0 | 3111.7 | 129.5 |
| 7 | 2776.9 | 140.0 | 2923.7 | 104.9 |
| 8 | 2776.9 | 140.0 | 2920.0 | 102.3 |
| 9 | 2776.9 | 140.0 | 2945.5 | 120.5 |

**Supplementary Table 8.** Recovery of isodehydrorhynchophylline.

| **No.** | **Content**  (μg·mL^-1^) | **Addition of isodehydrorhynchophylline**  (μg·mL^-1^) | **Measured content**  (μg·mL^-1^) | **Recovery**  (%) | **Average recovery**  (%) |
| --- | --- | --- | --- | --- | --- |
| 1 | 10757.1 | 8200.0 | 18859.6 | 98.1 | 102.7 |
| 2 | 10757.1 | 8200.0 | 18868.1 | 104.4 |
| 3 | 10757.1 | 8200.0 | 19049.6 | 105.4 |
| 4 | 10938.8 | 410.0 | 11286.1 | 106.9 |
| 5 | 10938.8 | 410.0 | 11320.7 | 74.2 |
| 6 | 10938.8 | 410.0 | 11435.4 | 129.5 |
| 7 | 10734.8 | 273.3 | 10969.2 | 104.9 |
| 8 | 10734.8 | 273.3 | 11065.9 | 102.3 |
| 9 | 10734.8 | 273.3 | 11062.0 | 120.5 |

**Supplementary Table 9.** Quantitative analysis of alkaloids in *Uncaria* ($\overline{\text{X}}$±SD, n=6).

| **Analyst** | **Content** (μg·g^-1^) |
| --- | --- |
| Rhynchophylline | 217.124±1.692 |
| Isorhynchophylline | 126.703±2.010 |
| Dehydrorhynchophylline | 55.182±0.670 |
| Isodehydrorhynchophylline | 215.823±2.541 |

**Supplementary Table 10.** Relative Standard Deviation **(**RSD) values of 10 ion signals of fatty acid species in negative ion modes.

| **Positive mode** (m/z) | ***t*_med_** (min) | **Instrument repeatability**  RSD (%, n=6) | **Method repeatability**  RSD (%, n=6) |
| --- | --- | --- | --- |
| 115.03752 | 0.56 | 1.42 | 2.35 |
| 180.06349 | 0.54 | 1.91 | 3.60 |
| 258.24119 | 7.45 | 3.11 | 4.00 |
| 304.23649 | 5.16 | 2.42 | 5.86 |
| 329.24893 | 5.61 | 2.33 | 7.25 |
| 403.30678 | 8.74 | 3.79 | 5.86 |
| 445.36913 | 14.06 | 2.55 | 3.22 |
| 562.48305 | 5.56 | 2.47 | 7.18 |
| 680.51523 | 16.06 | 2.92 | 1.34 |
| 511.47326 | 7.45 | 1.77 | 6.49 |

**Supplementary Table 11.** Relative Standard Deviation **(**RSD) values of 10 ion signals of lipid species in positive ion modes

| **Positive mode**  (m/z) | ***t*_med_** (min) | **Instrument repeatability**  RSD (%, n=6) | **Method repeatability**  RSD (%, n=6) |
| --- | --- | --- | --- |
| 286.22458 | 0.56 | 1.27 | 1.98 |
| 402.37264 | 0.54 | 4.98 | 2.19 |
| 465.45503 | 7.45 | 1.42 | 1.44 |
| 526.29276 | 5.16 | 0.91 | 0.72 |
| 657.59894 | 5.61 | 5.02 | 2.75 |
| 745.55770 | 8.74 | 1.13 | 1.31 |
| 754.56833 | 14.06 | 1.10 | 1.30 |
| 800.67310 | 5.56 | 1.06 | 1.42 |
| 832.66404 | 12.95 | 5.08 | 1.80 |
| 1498.08523 | 7.45 | 1.34 | 1.88 |

**Supplementary Table 12.** Relative Standard Deviation **(**RSD) values of 10 ion signals of lipid species in negative ion modes (n = 6)

| **Positive mode**  (m/z) | ***t*_med_** (min) | **Instrument repeatability**  RSD (%, n=6) | **Method repeatability**  RSD (%, n=6) |
| --- | --- | --- | --- |
| 159.98507 | 12.59 | 4.76 | 2.78 |
| 238.93127 | 11.29 | 2.67 | 4.31 |
| 306.91934 | 11.30 | 6.53 | 4.57 |
| 330.97062 | 0.94 | 3.46 | 9.18 |
| 480.30922 | 6.30 | 3.52 | 3.16 |
| 569.36468 | 6.23 | 5.18 | 3.13 |
| 626.91233 | 5.66 | 5.10 | 3.53 |
| 828.60191 | 6.23 | 8.05 | 7.00 |
| 961.62586 | 6.30 | 5.27 | 2.11 |
| 1091.72333 | 6.23 | 5.92 | 5.63 |

**Supplementary Table 13.** The full taxonomic names of all species used in manuscript

| Spices | Full taxonomic names |
| --- | --- |
| *Uncaria rhynchophylla* | *Uncaria rhynchophylla* (Miq.) Miq. ex Havil |
| *Semen raphani* | *Raphanus raphanistrum* subsp. sativus (L.) Domin |

**
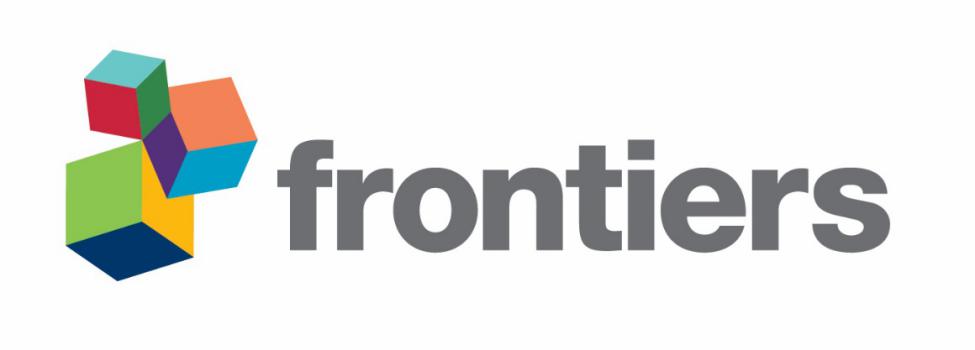
**
